# Impact of Physical Activity of Pregnant Women on Obstetric Outcomes

**DOI:** 10.3390/ijerph191912541

**Published:** 2022-10-01

**Authors:** Ksawery Goławski, Cezary Wojtyła

**Affiliations:** 1 Department of Obstetrics and Gynecology, Medical University of Warsaw, 02-015 Warsaw, Poland; 2 International Prevention Research Institute—Collaborating Centre, Calisia University, 16 Kaszubska St., 62-800 Kalisz, Poland

**Keywords:** physical activity, pregnancy outcomes, PPAQ, delivery, birthweight

## Abstract

Regular and well-planned physical activity (PA) has a positive impact on pregnancy outcomes. In this study, we determine the impact of the PA of pregnant women on the occurrence of certain pregnancy outcomes, such as type of labor, duration of pregnancy, and birthweight. The study is based on the results of a Polish national survey performed between 2011 and 2017 on a group of 9170 women. The Pregnancy Physical Activity Questionnaire (PPAQ) was used to estimate the PA of pregnant women. Light intensity PA accounts for the largest proportion of women’s total energy expenditure. Increase in women’s total energy expenditure was associated with an increase in the birthweight of a child. A similar relationship was observed in the case of light and moderate PA. Vaginal birth was more common among women with higher total energy expenditure. Mothers of preterm children showed lower energy expenditure for each type of PA compared to term pregnancies. There was also a correlation between moderate and vigorous PA and low birthweight. Our study indicates that PA undertaken by pregnant women has a positive impact on pregnancy outcomes.

## 1. Introduction

Regular physical activity during the pregnancy is one of the factors affecting its correct course [1]. The American College of Obstetricians and Gynecologists (ACOG) in its committee opinion from 2020 recommends at least 150 min of moderate-intensity aerobic activity per week during pregnancy and the postpartum period [2], consistent with WHO guidelines on physical activity and sedentary behavior [3]. Physical activity during pregnancy has a positive impact on the cardiovascular and musculoskeletal systems, which helps prevent conditions such as deep vein thrombosis and varicose veins of the lower limbs. Women undertaking physical activity during the pregnancy are less likely to suffer from lower limb swelling, muscle cramps, fatigue, shortness of breath, or osteoporosis in the future [4,5]. Preeclampsia is less common among physically active women due to the stimulation of the placenta for normal development and the prevention of pathological changes in its structure [6,7]. Gestational diabetes and pregnancy-induced hypertension are less likely as well. Physical activity, not only during pregnancy, is the main element in treating both of the above ailments [8,9]. Data indicate that undertaking physical activity improves the mood and psychological comfort of women in the postpartum period. Patients reporting higher energy expenditure during the second and third trimesters of pregnancy rated their quality of life as higher [10]. Among physically active pregnant women the frequency of postnatal depression is lower when compared to non-physically active women [11,12].

It is noteworthy that the impact of physical activity on the course of the pregnancy is dependent on the exercise intensity and duration. Regular and well-planned physical activity has a positive impact on the course of pregnancy and as a result the health condition of the newborn. Physical activity performed from the onset of pregnancy has a positive effect on the structure of the placenta, its surface area, and the number of vessels supplying it, which improves blood perfusion and thus its transport capacity [13]. However, the exercise needs to be performed reasonably. Both the lack of physical activity, as well as its excessive intensity, may result in creating detrimental conditions for the developing fetus. During high-intensity physical activity, disturbances of blood flow in the womb may occur. Submaximal-intensity exercise performed during the third trimester of pregnancy can cause a gradual increase in the systolic–diastolic index in the uterine artery. However, it is not observed in the umbilical artery [14]. A sedentary lifestyle, a stationary position for an extended time, but also intense exercise are all risk factors for the adverse pregnancy outcomes [15,16,17,18,19]. It is important to improve women’s awareness of the positive impact of physical activity on the pregnancy as studies show that both women of reproductive age and pregnant women increasingly limit their physical activity [20,21].

The study aimed to determine the physical activity of pregnant women in Poland and its impact on the occurrence of certain pregnancy outcomes such as type of labor, duration of pregnancy, and childbirth weight.

## 2. Materials and Methods

Analyses of the Caucasian population of pregnant women in Poland were carried out between 2011 and 2017 within the framework of the Polish Pregnancy-related Assessment Monitoring System organized and carried out by Chief Sanitary Inspectorate in Poland. This population-based study was carried out in all hospitals in Poland. A group of Polish women and their newborns were investigated during postpartum hospitalization (first days after delivery). The Ethics Committee, a body within the Institute of Rural Health in Lublin, approved of the study (reference number 03/2011).

All women in Poland who stayed in those hospitals whose director gave permission to carry out the survey were deemed eligible for the study. Informed consent was obtained from all women. Participation was voluntary. Thus, the study participants were those women who voluntarily agreed to fill in the survey during designated days of the study. The examination was carried out once in each hospital. The study was conducted simultaneously throughout the whole country, using the structures of the local Sanitary and Epidemiological Stations as units subordinate to the Chief Sanitary Inspectorate. These types of stations are located in every district in Poland, which allowed for efficient conduction of research throughout the country within one month of the year. In 2011 the study was conducted on one day in each hospital, during the third week of November and in 2012 during the third week of March. In 2017 the study was conducted between the 2 February and 22 March.

The Pregnancy Physical Activity Questionnaire (PPAQ) was used to estimate Physical Activity of pregnant women. PPAQ is form of survey validated for pregnant women and includes combinations of activities which reflect the physical activity (PA) patterns of the future mothers and allow us to isolate the specific PA types (household and caregiving, occupational, and sports activities). It also allows us to determine PA intensity (sedentary, light, moderate and vigorous), making it possible to establish a pattern of PA during pregnancy and comparison between various study populations. The 32 questions are related to everyday activities including household/caregiving activities (questions number 4–10 and 15–19), occupational activities (questions 32–36), as well as sport/exercise during leisure time (questions 23–31).

The respondents selected the category best estimating the amount of time spent on an activity per day or week. Time allotted to each activity from the questionnaire was then multiplied by its intensity. The intensity and the time predictor for each question were determined using the PPAQ instruction, based on the widely used compendium of physical activities, thus obtaining the energy expenditure measured in metabolic equivalent of task (MET) [22,23]. The result was then multiplied by the number of days in the week to measure the energy expenditure per hour per week (MET-h-week). Total PA of the pregnant women, classified by its intensity, was analyzed.

The methodology of this study and information about Pol-PrAMS are described in detail in a separate article [24].

### Statistical Analysis

Data were analyzed using IBM SPSS Statistics version 25.0 (IBM Corp., Armonk, NY, USA). The collected data, depending on variable type, were organized using descriptive analysis tables including sample size and percent or mean, median, standard deviation and values of the 25th and 75th percentile. The chi square test was used to analyze the dependency between categorical variables. The *p*-value of <0.05 for a two-tailed test was considered as statistically significant. During the newborn’s birth mass and pregnancy duration analysis, every of the physical activity types were categorized into the tertile groups. Linear regression analyses were conducted with consecutive measures of physical activity as the single independent variable and the number of completed weeks of gestation or birth weight of the newborn as dependent variables. The effect size measure for the linear regression analysis was R^2^. The adopted impact interpretation ranges for R^2^ were as follows: small ≤ 0.02; medium from >0.02 to ≤0.13; large > 0.13 [25].

For the purpose of the physical activity impact on the delivery method, the nonparametric Kruskal–Wallis test was used. If the result of the Kruskal–Wallis test was statistically significant, then the effect size associated with the Kruskal–Wallis analysis was calculated using the epsilon^2^ coefficient. For the epsilon^2^ coefficient the following effect size interpretation was adopted: small 0.01–<0.08; medium 0.08–<0.26; large ≥ 0.26 [26,27].

In order to evaluate the effect of physical activity on the delivery of the baby at less than 37 weeks’ gestation and less than 2500 g, the nonparametric Mann–Whitney test was implemented. The effect size in those cases was evaluated via Pearson correlation coefficient. In these cases, the following interpretation of the effect size was adopted (r): small ≤ 0.1; medium >0.1 ≤0.3; large > 0.3 [28].

In 2011, births took place in 393 hospitals in Poland, where 3682 mothers gave birth. The consent to perform the research was obtained from 379 directors of institutions, where 3082 mothers with newborn babies were hospitalized. Questionnaires from 2894 women were analyzed in our study. In 2012, 3555 mothers were hospitalized in 395 units during designated days for the study. The consent to conduct the study was obtained from the directors of 377 institutions, where 2905 mothers were hospitalized. A total of 2825 questionnaires were qualified in our study for the statistical analysis. In 2017, births took place in 397 hospitals and consent was obtained from 380 directors. A total of 3627 women were hospitalized and 3451 questionnaires were qualified for our statistical analysis. Overall, 9170 women took part in the study.

## 3. Results

### 3.1. Study Group Characteristics

Table 1 shows the characteristics of the study group of women. The highest percentage of women participating in the study was those 26 to 30 years old. This percentage was 36.9%. Comparable groups were women younger than 26 years old (25.6%) and 31–35 years old (26.2%). The smallest study group was women over 35 years of age.

The smallest study group consisted of women with primary education (6.5%), while the largest group consisted of women with higher education (47.5%), and an almost identical percentage consisted of women with secondary education (46.0%). Surveyed women lived mostly in the rural areas (42.0%). One-third of women lived in cities below 100,000 inhabitants, and almost one-fourth lived in large cities (above 100,000 inhabitants). More than half of women rated their social and living situation and material situation as good (55.2% and 59.1%, respectively). The social and living situation was described as poor by 15.6% of women, and the material situation by 27.9% of them. Most women were employed in white-collar jobs (44.6%). The unemployed accounted for 23.8% of the women and 5.1% were students. Just over 68% of women presented a normal body mass index, and 16.8% of the studied women were overweight and 6.1% suffered from obesity. Most women gave birth in a natural way (60.8%). In contrast, 38.2% of women gave birth by caesarean section. Assisted delivery (vacuum/forceps) took place in 1.1% of studied cases.

### 3.2. Physical Activity Energy Expenditure (MET-h/Week)

Physical activity of light intensity was declared by 96.35% of the studied women (8835/9170), moderate intensity by 96.12% (8814/9170), and vigorous-intensity by 85.93% (7880/9170), with 96.6% in total of the studied women (8862/9170) declaring undertaking physical activity during the pregnancy (Table 2).

It is noteworthy that there is a significant difference in total energy expenditure (MET-h/week) depending on exercise intensity. For the physical activity of light intensity, average energy expenditure was 94.98 MET-h/week, while the first quartile had an energy expenditure of less than 55.5 MET-h/week and the fourth quartile declared an energy expenditure of more than 124.4 MET-h/week. For moderate-intensity physical activity, medium weekly energy expenditure was 55.56 MET-h/week. For vigorous physical activity it was only 0.97 MET-h/week, while only 25% of studied women reported a weekly energy expenditure greater than 0 0 MET-h/week.

Considering total physical activity, average energy expenditure was 213.48 MET-h/week. In the first quartile, noted energy expenditure was lower than 135.1 MET-h/week, while in the fourth it was greater than 266.5 MET-h/week.

### 3.3. Physical Activity Impact on the Infant’s Birth Weight and the Duration of Pregnancy

Statistically significant impact of physical activity on the infant’s birth weight was observed in the following cases: total physical exertion (*p* = 0.038), as well as light exertion (*p* = 0.001), and moderate exertion (*p* = 0.02). The greater the total energy expenditure for each intensity of physical activity, the higher the average birth weight of infants. However, the size of the effect was not significant (R^2^ < 0.02).

Statistically significant impact of physical activity on the duration of pregnancy was noted for total physical exertion and light exertion (*p* = 0.006 and *p* = 0.008, respectively), the effect size was small as well (Table 3).

### 3.4. Pregnant Women’s Energy Expenditure (MET-h/Week) Impact on the Type of Labor

Pregnant women undertaking physical activity gave birth naturally more often. Statistically significant impact of physical activity on the type of labor was presented in each physical activity group (Table 4).

The effect size in all of the cases was small (epsilon from 0.001 to 0.002).

### 3.5. Pregnant Women’s Energy Expenditure (MET-h/Week) Impact on the Birth of the Child with the Birth Weight under 2500 g and before the 37th Week of Pregnancy

Impact of physical activity on the birth weight of the newborn under 2500 g turned out to be statistically significant in moderate and vigorous physical activity (*p*-value = 0.018 and 0.040, respectively). A low negative correlation coefficient was shown (−0.01) (Table 5).

Physical activity impact on the birth of the newborn before the 37th week of pregnancy was statistically significant in every physical activity group. The correlation coefficient was negative, with a slightly larger effect (−0.02 except for moderate-intensity physical activity). However, this is still not enough to concern it as a large effect size.

## 4. Discussion

The issue of pregnant women’s activity has become a relatively widely discussed topic in recent years, not only in the obstetrics and gynecology community. For both the mother and the infant, it has been shown to have a positive impact. It applies to both pregnancy and postpartum periods [29,30,31,32,33,34]. There are plenty of sources in the literature focused on the impact of physical activity on pregnancy and obstetrical results.

The size of the study groups varies significantly between studies. Many authors focus on physical activity during the pregnancy within the context of sports and performed exercises [29,35,36,37]. However, there is a lack of data evaluating physical activity in pregnancy within the wider context, understood as physical activity performed not only as a part of intentional exercises but also during everyday activities. There is a shortage of data analyzing physical activity in large groups of studied women, as well as large international meta-analysis that would allow for the complete analysis of the issue. In this article, the abovementioned issues were attempted to be solved in the analysis of the results from the 9170 studied women.

The characteristics of our study group presented consistency with studies addressing the issue of physical activity during pregnancy. Most of the patients were women with normal BMI (28.5–24.9 kg/m^2^), with secondary or higher education (in equal proportions), which corresponds with the studies of Hoffmann et al. or Kunath et al. [38,39]. The age of our patients did not seem to differ from the data found in the bibliography as well, given that 63.1% of this study group were in the 26–35 age range and the average age of patients in the bibliography oscillated around 30–31 ± 4 years old [32,33,40]. At the same time, there were examples of results significantly deviating from the values given above [34]; however, it cannot be ruled out that this may have been influenced by cultural differences [41,42]. The percentage of natural births did not deviate from the percentage of patients involved in similar research in the bibliography [32,39,43]. The clear deviation is perceivable only in the percentage of caesarean section delivery (38% in this study’s research vs. 28% in the bibliography). However, it is consistent with the fact that Poland has one of the highest rates of caesarean sections in Europe at 42.2% [44]. Regarding place of residence, a minority (42%) of women enrolled in the study come from rural areas, which corresponds with other studies based on questionnaires taken from postpartum women in Poland [45,46].

Within the context of weekly energy expenditure, based on the physical activity intensity, there was a noticeable downward trend—total energy expenditure of the studied women decreased with the increased intensity of the undertaken physical activity. This correlation was particularly noticeable in the case of vigorous-intensity physical activity, where average weekly expenditure was around 1 MET-h/week (for light and moderate-intensity physical activities, the values were several times higher). Hoffmann et al., in their research, describe similar phenomena [38]. What is different about this study, however, is that the standard deviation increases between light and moderate intensity activities, with the moderate-intensity activity even exceeding its mean value. This may be a necessity imposed by the need of a bigger engagement of pregnant women during higher intensity physical activity, which may escalate as the pregnancy progresses [47,48]. This is where potential differences in the frequency and regularity of undertaking a moderate-intensity physical activity, which is then reflected in absolute values for the entire study group, may come from. In the case of vigorous activity with such a small mean value, the standard deviation exceeding it (from a mathematical point of view) may not be that much of a surprise, and such a situation is in line with the study of Hoffmann et al. cited above [38].

In this study, a statistically significant positive impact of physical activity on the duration of pregnancy was demonstrated for total and light intensity physical activity. Jukic et al., in their prospective study (n = 1552), showed the statistical significance of a similar impact only for vigorous activity [49]. As mentioned before, we were unable to find, within the literature, other examples of studies analyzing the effect of physical activity intensity on the duration of pregnancy.

In the context of physical activity and its impact on the delivery method, Domenjoz et al. in their meta-analysis regarding case-control studies encompassing 3359 women showed that undertaking physical activity during pregnancy, statistically significant impacts the lowering of the incidence of the deliveries through caesarean section. Furthermore, the longer the total duration of the physical activity was, the greater the impact was [50]. In our study we demonstrated a statistically significant positive effect of physical activity on vaginal delivery, which was also reflected in the decreasing rate of caesarean sections between light and moderate intensity physical activity.

On the other hand, Barakat et al., based on their research, advocated for the lack of the impact of regular exercise on the delivery method [51]. Noteworthy is the high participation of primiparas in this study, whose deliveries are more likely to be complicated (especially in the first stage of delivery), which may result in the necessity to complete the pregnancy through caesarean section [52].

Unfortunately, there is little research in the literature that evaluates the impact of size and intensity of physical activity on the mode of delivery.

The impact of physical activity intensity on the statistically significant reduction of the low birthweights of newborns (<2500 g) was consistent with the literature, which describes similar phenomena [33,53,54]; however, other authors show no significant effect of physical activity on the risk of low birthweight newborns [55,56]. Hoffmann et al., in a multicenter study involving 2286 patients and depicting the impact of physical activity intensity on the preterm labor and neonatal birth weight, showed a statistically significant positive effect on the neonatal birth weight and the negative effect on the risk of preterm labor among women engaging in physical activity after 29 weeks of pregnancy. However, these correlations were not statistically significant after applying a division of physical activity by its intensity and then examining the impact of these individual categories [32]. On the other hand, Pastorino et al. in a large cross-sectional cohort analysis proved a small, but opposite, correlation between the physical activity of pregnant women and newborn birthweight, simultaneously confirming the authors’ results of decreased risk of preterm labor [55].

As evident herein, the results concerning the impact of physical activity during pregnancy on the birth mass are not conclusive. Some of the research exposes the positive impact [34,57], some negative [58,59], while others do not find any statistically significant correlation [50,60,61,62,63].

There is evidence of the inverted U-shaped association between PA and the birth mass [53]. Although this hypothesis is not without criticism [55], it largely explains the aforementioned discrepancies, as well as this study results in which statistical significance was proved in the case of light and moderate PA, as well as in the case of no category of physical activity intensity-total but not in the case of vigorous intensity.

For the impact of physical activity on the risk of preterm birth, there are many fewer discrepancies between the studies. D Aune et al., in their meta-analyses encompassing 20 randomized trials and 10 cohort studies, indicate that light and moderate physical activity during the pregnancy (vigorous intensity was not statistically significant) decreases the risk of preterm birth, which is largely consistent with our results [64].

There are limitations of this study that have been identified. First of all, the consensual nature of the study causes the risk that women agreeing to take part in it were more aware of their health, health behaviors, and their consequences. Thus, their physical activity and then obstetrical outcomes may differ from the general population.

Furthermore, the physical activity intensity scale is not identical in every study, which is due to the different tools used for its evaluation. Such circumstances may have an impact on the final results.

Finally, an incredibly significant issue is the credible and consistent evaluation of the intensity and energy expenditure of the performed physical activity. The questionnaire PPAQ used in this study is considered to be a reliable tool [65] able to be easily adopted in the region of its use [66,67,68,69,70]. Of course, it is not without its drawbacks—there is a risk of overestimating the amount of weekly physical activity [70,71]. The evaluation of the energy expenditure (MET-h) size is possibly not fully objective, underestimating the actual value in the case of physical activity among heavier and obese people [20]. Nevertheless, it seems difficult to find more optimal tool that can be reliably and relatively easily implemented in large-scale surveys.

## 5. Conclusions

Based on the study results, physical activity undertaken by pregnant women shows a positive impact on pregnancy outcomes. Physically active women gave birth naturally more often, their pregnancies lasted longer, and preterm births were less frequent. Newborn birth masses were higher, and the percentage of low birth masses was lower. Therefore, every obstetrician–gynecologist in her/his daily practice should encourage pregnant women to engage themselves in any form of physical activity, not only in intentional exercise. Such an attitude presents a health gain for everyone (mother and baby) involved in this extraordinary time of pregnancy.

## Figures and Tables

**Table 1:** Characteristics of the study population.

Feature	N	%	*p*
Age			<0.000001
≤25	2341	25.6	
26–30	3371	36.9	
31–35	2396	26.2	
>35	1023	11.2	
Total	9131	100.0	
Education			<0.000001
Primary	580	6.5	
Secondary	4081	46.0	
Higher	4213	47.5	
Total	8874	100.0	
Place of residence (inhabitants)			0.10
City (>100,000)	2225	24.7	
Town/city (<100,000)	3005	33.3	
Rural area	3784	42.0	
Total	9014	100.0	
Social conditions			<0.000001
Very good	2669	29.2	
Good	5043	55.2	
Average/poor	1425	15.6	
Total	9137	100.0	
Economic status			<0.000001
Very good	1190	13.0	
Good	5402	59.1	
Average/poor	2553	27.9	
Total	9145	100.0	
Working status			0.000003
Mental work	4053	44.6	
Physical work	2404	26.5	
Unemployed	2164	23.8	
Studying	462	5.1	
Total	9083	100.0	
BMI before pregnancy (kg/m^2^)			0.00014
<18.5	771	8.7	
18.5–24.9	6096	68.5	
25–29.9	1491	16.8	
≥30	543	6.1	
Total	8901	100.0	
Method of delivery			0.001
Vaginal	5396	60.8	
Caesarean section	3389	38.2	
Assisted delivery (vacuum/forceps)	95	1.1	
Total	8880	100.0	

**Table 2:** Physical activity energy expenditure (MET-h/week) classified according to its intensity.

Physical Activity	N	Mean	Median	S.D.	25th Percentile	75th Percentile
Intensity						
Light	8835	94.98	87.2	53.47	55.5	124.4
Moderate	8814	55.56	33.7	67.43	10.6	75.2
Vigorous	7880	0.97	0.0	3.42	0.0	0.0
Total PA	8862	213.48	193.3	119.22	135.1	266.5

**Table 3:** Impact of physical activity on infant birth weight and the duration of pregnancy.

Birth Weight (g)								Duration of Pregnancy (Weeks)						
Type of Physical Activity	N	Mean	S.D.	25th Percentile	75th Percentile	*p*-Value	R^2^	N	Mean	S.D.	25th Percentile	75th Percentile	*p*-Value	R^2^
Total						0.038	≤0.02						0.006	≤0.02
1st Tertile	2670	3340.04	560.90	3030	3700			2730	38.88	1.94	38.0	40.0		
2nd Tertile	2717	3374.84	571.58	3070	3740			2778	39.03	1.83	38.0	40.0		
3rd Tertile	2685	3379.09	576.59	3080	3750			2748	39.03	1.82	38.0	40.0		
Light						0.001	≤0.02						0.008	≤0.02
1st Tertile	2658	3328.92	565.91	3010	3700			2727	38.89	1.94	38.0	40.0		
2nd Tertile	2703	3384.02	565.79	3080	3750			2759	39.03	1.85	38.0	40.0		
3rd Tertile	2688	3381.88	575.03	3080	3750			2750	39.03	1.79	38.0	40.0		
Moderate						0.02	≤0.02						0.104	
1st Tertile	2663	3319.78	575.06	3000	3690			2718	38.90	1.96	38.0	40.0		
2nd Tertile	2686	3375.64	564.48	3100	3750			2752	39.03	1.84	38.0	40.0		
3rd Tertile	2676	3398.92	566.16	3100	3750			2741	39.01	1.79	38.0	40.0		
Vigorous						0.396							0.129	
1st Tertile														
2nd Tertile	5881	3360.76	571.73	3060	3720			6016	38.94	1.89	38.0	40.0		
3rd Tertile	1302	3390.84	545.88	3100	3750			1335	39.14	1.78	38.0	40.0		

**Table 4:** Impact of energy expenditure of pregnant women (MET-h/week) on the type of labor.

PA	Type of Labor	N	Mean	S.D.	Median	25th Percentile	75th Percentile	*p*	Epsilon^2^
Total	Vaginal	5237	217.6	122.1	195.9	137.8	269.0	0.022	0.001
	Caesarean section	3269	208.2	114.7	190.5	133.3	262.3		
	Assisted delivery	90	213.4	122.2	192.7	142.7	250.9		
Light	Vaginal	5223	96.9	54.2	88.7	56.4	126.7	0.001	0.002
	Caesarean section	3258	92.7	52.3	85.1	53.7	122.9		
	Assisted delivery	90	84.4	53.7	69.3	50.9	106.9		
Moderate	Vaginal	5216	58.0	70.2	34.9	11.4	77.9	0.001	0.002
	Caesarean section	3243	52.1	63.4	31.5	9.6	70.8		
	Assisted delivery	90	53.7	63.3	36.5	11.4	69.6		
Vigorous	Vaginal	4662	1.0	3.5	0.0	0.0	0.0	0.014	0.001
	Caesarean section	2900	0.9	3.2	0.0	0.0	0.0		
	Assisted delivery	82	1.2	3.7	0.0	0.0	0.0		

**Table 5:** Impact of energy expenditure of pregnant women (MET-h/week) on the birth of the child with the birth weight under 2500 g and before the 37th week of pregnancy.

			**<2500 g**							**<37 t.c.**						
<2500 g or <37 t.c.	Type of Physical Activity		N	Mean	S.D.	25th Percentile	75th Percentile	*p*-Value	Effect Size	N	Mean	S.D.	25th Percentile	75th Percentile	*p*-Value	Effect Size
	Total							0.530							0.021	−0.02
0		≥	7596	213.95	118.19	136.0	266.2			7630	214.36	118.18	136.4	266.8		
1		<	479	212.45	130.10	129.4	268.7			626	207.07	128.04	125.3	263.0		
	Light							0.153							0.007	−0.02
0		≥	7576	95.43	53.09	56.0	124.3			7614	95.50	52.99	55.8	124.3		
1		<	476	93.67	59.41	49.1	128.2			622	91.00	57.95	50.4	123.6		
	Moderate							0.018	−0.01						0.009	−0.01
0		≥	7554	55.73	67.27	10.6	75.3			7591	55.81	67.53	10.6	75.3		
1		<	474	53.01	69.63	8.0	71.4			620	53.18	69.68	8.0	73.1		
	Vigorous							0.40	−0.01						0.001	−0.02
0		≥	6768	0.95	3.35	0.0	0.0			6801	0.97	3.40	0.0	0.0		
1		<	418	0.77	2.98	0.0	0.0			550	0.72	2.82	0.0	0.0		

## Data Availability

Not applicable.
